# Giant idiopathic penoscrotal lymphedema - Surgical approach with skin graft: A case report^☆^

**DOI:** 10.1016/j.eucr.2022.102208

**Published:** 2022-08-30

**Authors:** Raphael Flavio Fachini Cipriani, Leonardo Fleury da Silva, Maitê Mateus, Ivam Vargas Martins da Silva, Renato da Silva Freitas, Rodrigo Ketzer Krebs

**Affiliations:** Universidade Federal do Paraná, Hospital de Clínicas, Curitiba, Brazil

**Keywords:** Lymphedema, Reconstructive surgery, Scrotum

## Abstract

Lymphedema is originated from the reduced lymphatic flow, causing a volumetric increase in the affected region and is physically and emotionally uncomfortable. Surgical intervention is considered the best treatment option as it brings both functional and aesthetic benefits. This is a report from the case of a previously healthy 45-year-old man who developed idiopathic penoscrotal giant lymphedema. A surgical approach was proposed. There was resection of scrotal lymphedema followed by a classic postectomy, suprapubic advancement flaps, and use of a partial skin graft from the right thigh. The results of the therapeutic approach were aesthetically and functionally satisfactory.

## 1 Introduction

Lymphedema is accumulation of lymph fluid, as a consequence of reduction in lymphatic flow, promoting a volumetric increase. The genital form is the 3rd most frequent.^1^ Lymphedema may have an incidence of up to 20% in tropical countries, but it is uncommon in clinical practice. It is classified, according to its cause, as primary or idiopathic, due to obstruction, malformation or hypoplasia of lymphatic vessels, or secondary, more frequent, associated with acquired dysfunctions and lymphatic obstruction.^2^

Malignancy and filariasis are the main causes. Other etiologies are lymphogranuloma, mycobacterium spp. and cellulitis. Benign tissue lymphoproliferation causes massive lymphoedema, mainly associated with obesity. The disease is physically and emotionally uncomfortable, being disabling because of pain, difficulty in hygiene, mobility, social and sexual impairment.^2^

Conservative treatment is decompressive and usually the first line. However, the results are temporary and recurrence is frequent. Therefore, surgical intervention is considered the best treatment option. The technical possibilities aim to increase drainage capacity or remove affected areas in cases of fibrosis or fatty infiltration and consists in excision of the affected tissue with primary closure, or reconstruction with grafts and flaps. This approach brings functional and aesthetic benefits and improving quality of life.^2^

## 2 Case presentation

**Fig. 1:**
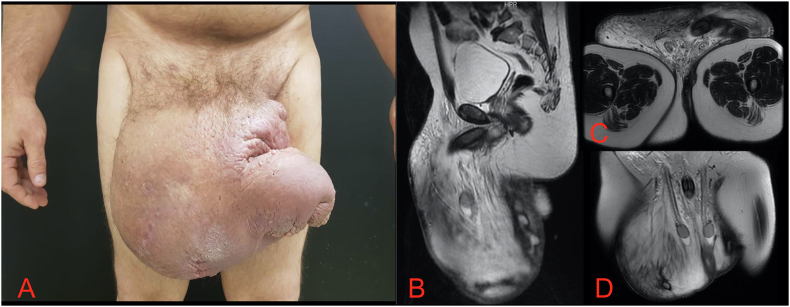
A: Clinical presentation of giant penoscrotal lymphedema; B: Sagittal section of MRI; C: Axial section of MRI; D: Coronal view of MRI with increased testicular pouch volume, diffuse subcutaneous edema and scrotal wall thickening.

AEK, male, 49 years, was referred to the urology service of a Tertiary Hospital, complaining of a progressive and voluminous penoscrotal increase for 6 years. In 2012, he presented a small lesion in the scrotum. It evolved with subsequent recurrent drainage (2014) and fluctuating edema. He denied comorbidities or previous surgeries and was a smoker. Physical examination revealed giant penoscrotal lymphedema (Fig. 1). Resonance imaging (MRI) detected a marked increase in the volume of the scrotum secondary to diffuse subcutaneous edema and thickening of the scrotal wall, compatible with lymphedema and inflammatory process in the skin of the testicular pouch (Fig. 1). During the investigation, no secondary causes were identified, being classified as idiopathic.

There was a functional repercussion, with urinary and hygiene difficulties, low sexual performance, psychological impact, due to impaired aesthetics, and no response to clinical treatment.

**Fig. 2:**
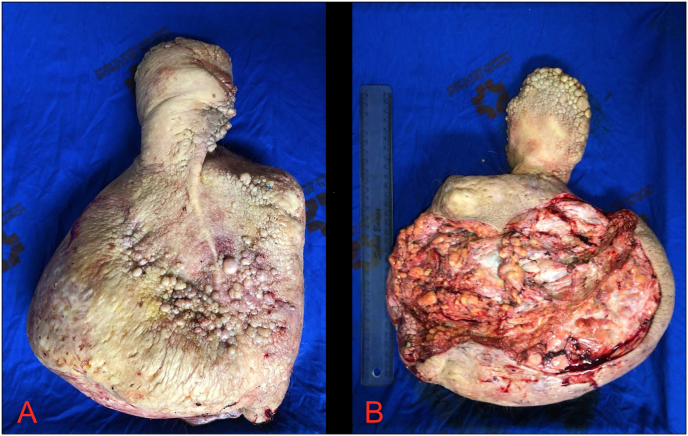
Surgical specimen from the excision; A: ventral view and B: dorsal view.

The whole area of large lymphedema proportions has been surgically removed from the inguinal region, isolating the spermatic funiculus and testicles, and dissecting the penis in anterograde way, preserving dorsal vascularization (Fig. 2). For the scrotum defect correction, advancement of skin flaps were used, from the posterior perineal region, which was not affected by lymphedema. For the penis coverage, a partial skin graft was used, whose donor area was the internal face of the patient's right thigh. The use of tissue affected by lymphedema was avoided, because it is a thick and rigid skin, making it difficult to capture a thin partial skin graft by the Blair blade. Thin tissue grafting enables better integration, aesthetic and functional results and reduced graft loss.

**Fig. 3:**
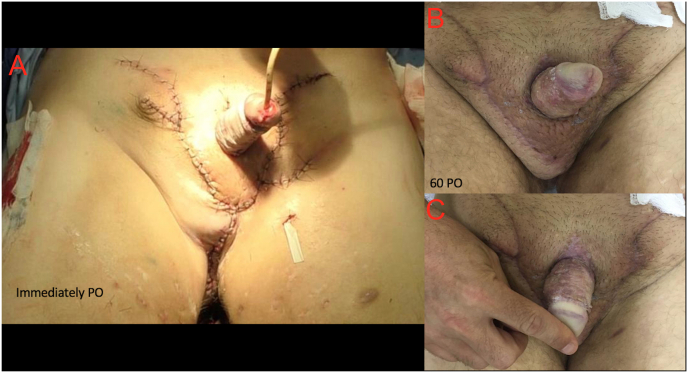
A: Results in the immediate postoperative; B and C: Results in the 60 P.O.

The procedure was uneventful. The patient was discharged from the hospital on the sixth postoperative day. During follow-up, the bladder catheter and stitches were removed 8 and 23 days after surgery, respectively. On the 60th postoperative day (Fig. 3), the recovery of the regional anatomy, improved esthetics and good healing was evident. The patient reported improvement in sexual, hygienic and voiding functionality. There were no postoperative complications, probably due to the use of a posterior perineal flap and healthy skin graft, with a long recurrence-free period.

## 3 Discussion

There is no cure for lymphedema, but the identification and adequate choice of a technique allow its management and better quality of life, with a long time free from remission. Usually, the first line is conservative decompression treatment. At first, it may appear to have good results, but are usually temporary. Surgery is more effective, especially in relapsing, refractory or giant lymphedemas, as described.

Delpech first described the reconstruction with thigh flaps. Possibilities are closure by edges approximation, grafting in the penis and/or scrotum, flaps rotation for the scrotum, or even skin from the excised tissue itself as a graft - not generally used, because of the thickening. Excision is the most described surgical procedure^2^

The systematic review by Aulia et al. identified that all studies demonstrated quality of life improvement after intervention, with better subjective perspective, mobility, urinary function, sexual activity and scrotal reduction. The reported case had all of these aspects upgraded until now.

Scaglioni and Uyulmaz performed a lymphedema excision (debulking) followed by a partial skin graft (upper thigh) for the penis. There were no recurrences or complications within six months of follow-up. Similarly, in this case, there has not been recurrence so far.^3^

Some early complications highlighted by Torio-Padron et al. were hematoma (4%) and dehiscence (2%). In these patients, preoperative decongestive physiotherapy and reduction surgery were used.^4^ The follow-up time was not clarified and, unlike Scaglioni and Uyulmaz, Torio Padron did not use grafts or flaps in his patients. However, even with the complications there was significant improvement in physical well-being and general functions. Other complications reported are cellulitis and infections. Furthermore, the earliest relapse was in the second year after the intervention.^5^

In the described report, grafts and flaps were also used and, equally, there were no complications. This suggests there seems to be less identification of recurrences and complications when surgical techniques with grafts and flaps are used. However, epidemiological studies with larger samples are needed, in addition to specific cases, to establish such conclusions.

## 4 Conclusions

Giant penoscrotal lymphedema is uncommon but substantially detrimental to the patient's quality of life. Usually associated to filariasis, but can be present idiopathically. Bibliography shows that clinical measures are ineffective and uncomfortable, specifically in genital region.

Therefore, surgical management with the removal of the affected tissue and genital reconstruction is the method of choice for the best recovery of the physical and emotional capacities of affected patients - especially because of aesthetic and functional recovery, as demonstrated in this case report, as well as a low rate of complications - possible when an adequate surgical technique is performed, by a prepared team, using grafts and flaps, postoperative care and outpatient follow-up.
